# Kidney transplantation in mitochondrial diseases: a systematic review

**DOI:** 10.1007/s00467-025-07034-3

**Published:** 2025-11-27

**Authors:** Sze Wa Wong, Cheuk Wing Fung, Fred Tomlin, Jelena Stojanovic

**Affiliations:** 1 Paediatric Nephrology Centre, Hong Kong Children’s Hospital, Kowloon City, Hong Kong SAR; 2 Metabolic Medicine, Hong Kong Children’s Hospital, Kowloon City, Hong Kong SAR; 3 Department of Paediatric Nephrology, Great Ormond Street Hospital for Children NHS Trust, London, UK; 4 Institute for Child Health University College London, London, UK

**Keywords:** Kidney failure kidney transplantation, Primary mitochondrial diseases, Patient survival

## Abstract

**Background:**

Primary mitochondrial diseases are a group of rare, heterogeneous, multisystem disorders. While renal involvement is increasingly recognised, especially in paediatric patients, data on kidney transplantation outcomes in this population remain limited.

**Objectives:**

To evaluate kidney transplantation outcomes in genetically confirmed primary mitochondrial diseases with multi-organ involvement and provide clinical insights from systematic literature review.

**Data sources:**

We systematically searched PubMed, MEDLINE, EMBASE and Google Scholar from inception to 10 June 2025 using keywords and MeSH terms related to “mitochondrial disease”, “transplantation” and “outcome”.

**Study eligibility criteria:**

We included studies that reported post-transplant clinical outcomes in patients with genetically confirmed primary mitochondrial diseases. Studies without genetic confirmation or transplant follow-up were excluded. Patients with Co-enzyme Q 10 deficiency were excluded as they mainly manifest as isolated steroid resistant nephrotic syndrome with subtypes that respond well to co-enzyme replacement.

**Participants and interventions:**

Participants included paediatric or adult patients diagnosed with genetically confirmed primary mitochondrial diseases who received isolated kidney transplant from living or deceased donor.

**Study appraisal and synthesis methods:**

Data were extracted on demographics, genotypes, renal and extra-renal features, transplant characteristics, complications and outcomes. Risk of bias was assessed qualitatively by two independent reviewers. Discrepancies were resolved through consensus or discussion with third reviewer. Due to clinical and methodological heterogeneity, a narrative synthesis was performed.

**Results:**

Forty-six patients (15 paediatric, 31 adult) were included from 18 eligible studies. Ten patients had *RMND1*-related disease. All harboured either homozygous or compound heterozygous *c.713A* > *G* variants in *RMND1*. Thirty patients carried the *m.3243A* > *G* mtDNA point mutation variant in *MT-TL1*. The remaining six patients harboured an *m.3271 T* > *C* variant in *MT-TL1*, single mtDNA deletions, *m.8618dup* in *MT-ATP6*, *m.12418delA* in *MT-ATP6* and *m.13513G* > *A* in *MT-ND5* respectively. At nephrology referral, chronic kidney disease and kidney failure each was present in 26.1% of patients. Median time from renal presentation to kidney failure was 6 years. Graft and patient survival exceeded 90% across different genetic mutations and age groups. Post-transplant deterioration of neurological or metabolic features was reported predominantly in patients with an *m.3243A* > *G* variant.

**Limitations:**

The review is limited by small sample size, selection and reporting bias, heterogeneous follow-up durations and outcome measures. Data were derived mainly from case reports and small case series.

**Conclusions and implications of key findings:**

Kidney transplantation is a viable option of kidney replacement therapy for patients with mitochondrial diseases. Patients with primary mitochondrial diseases should be considered for kidney transplantation. Further prospective studies are needed to define optimal transplant timing, immunosuppression strategies and long-term systemic outcomes.

**Systematic review registration number:**

CRD420251086889.

**Graphical abstract:**

**Figure:**
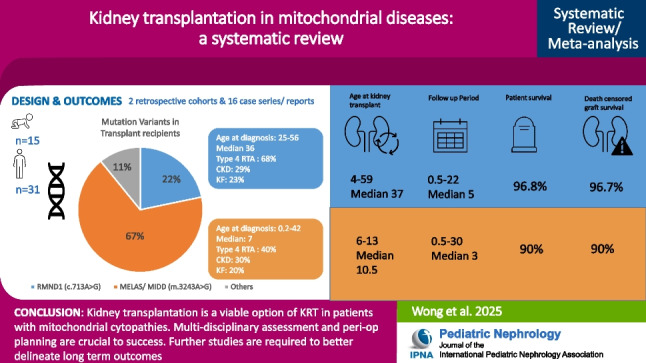
A higher resolution version of the Graphical abstract is available as Supplementary information

**Supplementary Information:**

The online version contains supplementary material available at 10.1007/s00467-025-07034-3.

## Introduction

Primary mitochondrial diseases are a heterogeneous group of rare disorders resulting from dysfunction of the mitochondrial respiratory chain. These conditions often involve multiple organ systems and typically present with non-specific early symptoms such as fatigability or weakness, which pose significant diagnostic challenges [1]. Commonly encountered mutations with predominant kidney involvement include mitochondrial DNA (mtDNA) point variants in the *MT-TL1* gene, which can cause Mitochondrial Encephalomyopathy, Lactic Acidosis and Stroke-like episodes (MELAS) or Maternally Inherited Diabetes and Deafness (MIDD); *MT-ATP6* gene mutations associated with Leigh syndrome; nuclear DNA (nDNA) variants such as *RMND1*; and primary Co-enzyme Q (CoQ) 10 deficiency, due to mutations in CoQ biosynthesis genes, e.g. CoQ 2, 4, 6 and 8B mutations [2, 3]. The estimated prevalence is 4.7 per 100,000 in children and 1 per 4300 in adults [4, 5]. This discrepancy likely reflects both genotype distribution and disease course: nuclear gene defects such as *RMND1* typically present in early childhood with severe manifestations and high mortality, whereas mtDNA variants such as *m.3243A* > *G* usually have a more insidious onset, predominating in adult series [4, 6]. Since the first description by Luft et al. in 1962 [7], more than 350 mtDNA and nDNA variants have been linked to an expanding spectrum of clinical syndromes [3, 8, 9].

Renal involvement is reported in 5–25% of individuals with mitochondrial disease [1, 3–14]. The age of onset and clinical severity vary widely, depending on the causative mutation and, in mtDNA disorders, on heteroplasmy levels. Proximal tubulopathy is the most frequent renal presentation, but some patients develop nephrotic-range proteinuria and steroid-resistant nephrotic syndrome (SRNS), or progress rapidly to kidney failure within the first two years of life [3, 15, 16]. Historically, paediatric mortality rates reached 70%, with a median age of death as early as 13 months [17, 18]. However, advances in genetic diagnostics and multidisciplinary care over the past two decades have improved survival, with more children now living into later childhood with stage 2 or above chronic kidney disease (CKD). A multicentre study by Parasyri et al. reported CKD in up to 75% of patients with primary mitochondrial diseases [19]. Kidney failure in early childhood is also being reported with increasing frequency [20–22].

As survival improves and more children with primary mitochondrial disease progress to advanced CKD, understanding the outcomes of kidney transplantation has become increasingly important. Nevertheless, the evidence remains limited to case reports and small case series. This review addresses this gap by synthesising published data on kidney transplantation in primary mitochondrial diseases. We focus on progressive, multisystem disorders characterised by irreversible kidney damage, for which supportive care and kidney transplantation are the mainstays of management. We excluded CoQ 10 deficiency because it presents as isolated SRNS and its subtypes, like CoQ4 variants, respond favourably to oral enzyme replacement, which can reduce proteinuria or even reverse kidney impairment [10–12, 23, 24]. To preserve clinical coherence and avoid over-estimating transplant outcomes with conditions responsive to specific therapy, we prespecified the exclusion of primary CoQ biosynthesis defects from our synthesis. Nonetheless, CoQ-related disorders remain relevant to transplant decision-making; we provide a contextual discussion of their renal phenotypes and outcomes.

## Method

### Search strategy

A systematic literature search was conducted across PubMed, MEDLINE, EMBASE and Google Scholar from database inception to 10 June 2025. Search terms included combinations of keywords and Medical Subject Headings (MeSH) related to “mitochondrial disease”, “kidney transplantation” and “clinical outcomes” (see Supplemental Fig. 1).

**Fig. 1:**
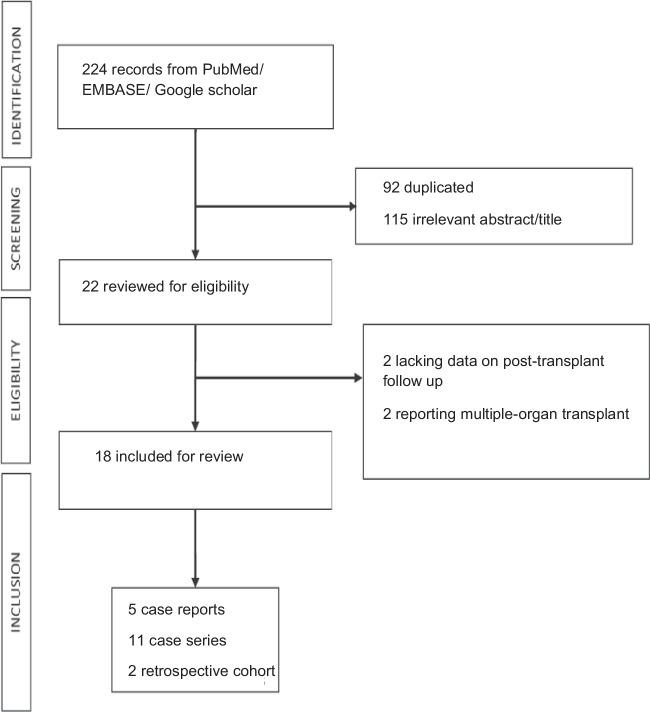
PRISMA flow chart of study identification

### Inclusion/exclusion criteria

Only peer-reviewed, full-text articles reporting post-transplant clinical outcomes in genetically confirmed mitochondrial disease were included. Grey literature, conference abstracts and unpublished studies were excluded. Both adult and paediatric cohorts were eligible for inclusion. Studies lacking genetic diagnosis or post-transplant follow-up data were excluded. Studies reporting multiple solid organ transplantation were excluded, as our focus was on kidney transplantation outcomes. Also, including these patients could have introduced confounding in the survival analysis. CoQ deficiencies were excluded, as described in the Introduction, due to their distinct clinical profile and therapeutic response.

### Screening and bias assessment

Two independent reviewers screened titles and abstracts and assessed full texts for eligibility. For case reports or series, the risk of bias and methodological quality were assessed by the reviewers independently based on principles described by Murad et al. [25]. For retrospective cohorts, BMJ AXIS Tool for Observational Cohort and Cross-Sectional Studies was used [26]. Discrepancies were resolved by consensus or discussion with a third reviewer.

### Data extraction

Data were extracted from eligible publications on study design, patient demographics, genetic diagnosis, renal and extra-renal features, age at kidney transplantation, donor type, peri-transplant complications, immunosuppression, post-transplant disease progression, graft function, patient survival and conflicts of interest.

### Data synthesis

Because of the clinical and methodological heterogeneity of identified publications, essentially a narrative synthesis was performed instead of statistical synthesis. Categorical variables, e.g. presence of neurological features and type of genetic mutation, were summarised as counts and percentages. Continuous variables, such as age at diagnosis and time to kidney failure, were expressed as medians with ranges. Categorical variables were analysed using the chi-square test, while continuous variables were compared using the Kruskal–Wallis test with post hoc pairwise comparisons when appropriate. A *p*-value < 0.05 was considered statistically significant. When patient-level data were missing or inconsistently reported, they were excluded from specific subgroup analyses but included in the overall descriptive synthesis.

The review was registered in PROSPERO (registration number: CRD420251086889) and the protocol is available online.

## Results

A total of 229 articles were identified through database searches. After removing 92 duplicates, 115 were excluded based on title and abstract screening for irrelevance. Twenty-two full-text articles were assessed for eligibility. Of these, two were excluded due to lack of post-transplant clinical data, another two were excluded because they involved multi-organ transplantation (Supplemental Table 1). Ultimately, 18 studies were included in the review. The PRISMA flowchart outlining study selection is shown in Fig. 1. Included studies comprised two retrospective cohort, 11 case series and five case reports, of which the quality assessment is listed in Table 1 [20, 22, 27, 28]. Each retrospective cohort was rated as “Good” quality. Of the 16 case reports/series, 6 were considered “Good”, while 8 and 2 were “Fair” and “Poor” respectively. Individual quality assessment is summarised in Supplemental Tables 2A&B.

**Table 1:** List of included publications for review

	**Article type**	**Countries**	**Quality assessment**
Ravn et al. [20]	Case series	Denmark	Fair
Roper et al. [22]	Case series	UK	Fair
Hameed et al. [27]	Case report	UK	Good
Lederer et al. [29]	Case report	Germany	Good
Johnson et al. [30]	Case series	France	Good
		USA	
Laat et al. [31]	Case series	The Netherlands	Good
Szczepanik et al. [32]	Case report	USA	Fair
Ducharlet et al. [33]	Case report	Australia	Fair
Sousa et al. [34]	Case report	Brazil	Good
Guery et al. [35]	Case series	France	Poor
Nishida et al. [36]	Case series	Japan	Good
Parikh et al. [37]	Multi-centre	Australia	Good
	Retrospective	Canada	
	Cohort	USA	
		UK	
Broenen et al. [38]	Case series	France	Fair
Shayota et al. [39]	Case series	USA	Fair
Kömhoff et al. [40]	Case series	Germany The Netherlands	Fair
Ng et al. [41]	Multi-centre	Australia	Good
	Retrospective	Czech	
	Cohort	Denmark	
		Ireland	
		Italy	
		Spain	
		Pakistan	
		UK	
		USA	
Seidowsky et al. [42]	Case series	France	Poor
Stein et al. [28]	Case series	UK	Fair

Forty-six patients were included in the review, comprising 15 paediatric and 31 adult recipients (Table 2). Individual-level clinical data are presented in Supplemental Table 3.

**Table 2:** Summary of patients with different genetic variants from previous literature

	RMND1	(*n* = 10)	MELAS/MIDD	(*n* = 30)	Others	(*n* = 6)	
Mutation type and variants	Nuclear mutation		mtDNA point mutation		mtDNA deletion	(*n* = 2)	
	*c.713A* > *G*		*m.3243A* > *G*		mtDNA point mutations: m.8618dup m.12418delA m.13513G > A *m.3271 T* > *C*	(*n* = 4)	
Sex (M:F)	3:7		15:16		3:2		
Median age at first presentation	0.75 (range 0.1–2)		22 (range 5–36)		2 (range 0.3–26)		*p* < 0.05
Median age at diagnosis	7.25 (range 0.2–42)		36 (range 25–56)		23 (range 2–38)		*p* < 0.05
**Extra-renal manifestations before transplant**							*p* < 0.05
1. Neurological (overall)	10	100.0%	27	90.0%	5	100.0%	
- SNHL	9	90.0%	23	76.7%	4	80.0%	
- Developmental delay	7	70.0%	0	0.0%	1	20.0%	
- Hypotonia	6	60.0%	0	0.0%	0	0.0%	
- Stroke	0	0.0%	5	16.7%	0	0.0%	
- Myopathy	1	10.0%	5	16.7%	1	20.0%	
- Cognitive impairment	0	0.0%	4	13.3%	2	40.0%	
- Others	2	20.0%	5	16.7%	1	20.0%	
2. Faltering growth	9	90.0%	3	10.0%	1	20.0%	
3. Endocrine	1	10.0%	18	60.0%	1	20.0%	
4. Cardiovascular	4	40.0%	7	23.3%	1	20.0%	
5. Others	0	0.0%	3	10.0%	1	20.0%	
**Renal manifestations**							
Median age at first presentation to nephrologists	5 (range 0.25–12)		31 (range 5–44)		23 (range 6–31)		*p* < 0.05
Type of presentation							
1. Nephrotic syndrome	3	30.0%	3	10.0%	1	20.0%	*p* < 0.05
2. Nephrotic range proteinuria	0	0.0%	18	60.0%	0	0.0%	
3. Renal Fanconi	0	0.0%	0	0.0%	1	20.0%	
4. CKD	3	30.0%	9	30.0%	0	0.0%	
5. Kidney failure	2	20.0%	6	20.0%	3	60.0%	
6. type 4 RTA	4	40.0%	0	0.0%	0	0.0%	
7. Others	0	0.0%	1	3.3%	0	0.0%	
**Histology**		(*n* = 3)		(*n* = 20)		(*n* = 1)	N/A
FSGS	1	33.3%	15	75.0%	1	100%	
TIN and IFTA	2	66.7%	1	5.0%	0		
Others	0	0.0%	4	20.0%	0		
Median age at KF	10.5 (range 5–13)		37 (range 15–58)		23 (range 6–31)		*p* < 0.05
Median time from renal presentation to KF	6 (range 2–8)		6 (range 1–22)		1		
**Kidney transplantation**							
Median age at transplant	10.5 (range 6–13)		39 (range 4–59)		23 (range 8–32)		*p* < 0.05
Acute peri-operative complication	0	0.0%	0	0.0%	1	20.0%	
Mitochondrial disease–related complications	0	0.0%	1	3.2%	0		
Hyperacute rejection	0	0.0%	0	0.0%	0		
**Outcome**							
Median follow-up period	3.25 (0.5–30)		5 (0.5–22)		5 (range 1–10)		
New-onset/worsened comorbidities post-transplant							
- Neurology	0	0.0%	6	19.4%	3	60.0%	
- Cardiovascular	0	0.0%	4	12.9%	0	0.0%	
- Endocrine (new-onset DM/worsened control)	0	0.0%	10	32.3%	1	20.0%	
- Others	0	0.0%	2	6.5%	0	0.0%	
Rejection							
- T cell rejection	1	10.0%	2	6.5%	1	20.0%	
- Antibody mediated rejection	0	0.0%	0	0.0%	1	0	
**Viremia**	0		1	3.2%	0	0	
Death-censored graft survival	9	90.0%	29	96.7%	5	100.0%	*p* 0.76
Patient survival	9	90.0%	30	96.8%	5	100.0%	*p* 0.58

Ten patients harboured pathogenic variants in *RMND1*, a nuclear gene, all carrying the variant c.713A > G in either homozygous or compound heterozygous state. Thirty patients carried the *m.3243A* > *G* mtDNA point mutation in *MT-TL1*. Among the remaining six recipients, two had single large-scale mtDNA deletions, one carried *m.8618dup* in *MT-ATP6*, one carried *m.3271 T* > *C* in *MT-TL1*, one carried *m.12418delA* in *MT-ATP6* and one carried *m.13513G* > *A* in *MT-ND5*.

In patients with *c.713A* > *G* variants in *RMND1*, the median ages at first presentation and diagnosis were 9 months and 7 years, respectively. Indeed, all presented at or before age 2. In contrast, 77.4% of patients having an *m.3243A* > *G* point mutation in *MT-TL1* presented in adulthood (median age 22 years) and were diagnosed at a median age of 36 years.

Renal manifestations varied by genotype. Most patients with an *m.3243A* > *G* variant in *MT-TL1* (70.0%) presented with nephrotic-range proteinuria or nephrotic syndrome at a median age of 31 years (range 5–44). Patients with *c.713A* > *G* variants in *RMND1* presented earlier (median 5 years; range 0.25–12) with more heterogeneous kidney involvement: 40% developed type 4 renal tubular acidosis (RTA), with or without hypertension, 30% had CKD at various stages and 20% had kidney failure at first nephrology review. Excluding those already in kidney failure at presentation, the time from first renal presentation to kidney failure ranged from 2 to 8 years (median 6 years) in these patients with a median age at kidney failure of 10.5 years. In those with point mutation *m.3243A* > *G* in *MT-TL1*, kidney failure developed over 1–22 years (median 6 years) at ages between 15 and 58 years (median 37).

Extra-renal features before transplantation were common. Neurological manifestations were present in 90.0–100.0% of patients across different genotypes. Sensorineural hearing loss occurred in 66.7–90.0%. Developmental delay, as reported in paediatric patients, affected 70.0% of those with *RMND1* mutations. Patients with *m.3243A* > *G* mutation in *MT-TL1* had more heterogeneous neurological complications, including stroke (16.7%), myopathy (16.7%) and cognitive impairment as described in adults (13.3%). Faltering growth (90%) and cardiovascular involvement (40%) were significantly more frequent in *RMND1* mutation (*p* value < 0.05).

The median age at kidney transplantation ranged from 10.5 to 39 years, with median follow-up durations of 3–5 years. Early post-operative complications were rare: one patient carrying an *m.3243A* > *G* variant in *MT-TL1* developed pseudo-obstruction, and another with mtDNA deletion developed posterior reversible encephalopathy syndrome (PRES); both resolved with supportive care.

Death-censored graft survival and patient survival did not differ significantly between genetic subgroups (*p* = 0.58 and 0.57, respectively). Death-censored graft survival rates were 90% (9/10) in patients with *RMND1* mutation, 96.6% (28/29) in *m.3243A* > *G in MT-TL1* and 100% (6/6) in other variants. Corresponding patient survival rates were 90% (9/10), 96.8% (29/30) and 100% (6/6). One patient carrying an *RMND1* mutation lost graft function due to a well-recognised perioperative transplant risk, arterial thrombosis 4 weeks post-transplant, resumed haemodialysis and died of recurrent bacteraemia 2 years later. One patient with mtDNA deletion experienced T cell–mediated rejection (TCR) and responded to medical treatment without graft loss. Among patients with the *m.3243A* > *G* variant in *MT-TL1*, two (6.7%) developed TCR with graft function preserved following medical treatment; one died of recurrent stroke and complications 2 years after transplantation; another developed graft failure 5 years after receiving a kidney from her mother, who unknowingly carried the same *m.3243A* > *G* variant. Her graft biopsy confirmed recurrence of focal segmental glomerulosclerosis (FSGS).

Post-transplant disease progression involving extra-renal systems was frequent in *m.3243A* > *G* in *MT-TL1* (70.0%, 21/30). One-third developed New-onset Diabetes After Transplant (NODAT) or worsening glycaemic control; 16.7% (5/30) had neurological deterioration, mainly worsening myopathy; and 13.3% (4/30) developed hypertrophic or dilated cardiomyopathy. Two patients with mtDNA deletion continued to experience cognitive and motor decline. One patient with *m.8618dup* in *MT-ATP6* developed psychosis, myopathy and NODAT. No post-transplant extra-renal deterioration was documented in patients with *RMND1* mutation. Overall, these patients suffered from standard risks and complications associated with kidney transplant such as thrombosis and rejection.

Stratified by age, the majority of paediatric patients (60%) carried *c.713A* > *G* variants in *RMND1* gene, whereas most adults (90%) harboured the *m.3243A* > *G* variant in *MT-TL1*. Given this genotype predominance, the results stratified by age mirrored those stratified by genotype. Both paediatric and adult patients progressed to kidney failure relatively rapidly, with a median interval of 4–6 years from first presentation. In terms of outcomes, death-censored graft survival was 93.3% (14/15) in the paediatric group and 96.7% (29/30) in adults, while patient survival was 93.3% (14/15) and 96.8% (30/31), respectively (Tables 3 and 4). Overall, graft and patient survival rates were broadly comparable between paediatric and adult recipients.

**Table 3:** Summary of paediatric patients identified from previous literature

Demographics	(*n* = 15)	
Sex	M:F 46.7%:53.3%	7:8
Median age at first presentation (years) (*n* = 11)	0.75	(range 0.1–5)
Median age at diagnosis of mitochondrial diseases (*n* = 8)	1	(range 0.2–15)
Mutations		
mtDNA mutation		
- m.3243A > G	20.0%	
- mtDNA deletion	13.3%	
- m.12418delA	6.7%	
Nuclear gene mutation		
- c.713A > G (homozygous/compound heterozygous)	60.0%	
Phenotypes		
RMND1-related diseases	60.0%	9
MELAS	33.3%	5
Kearns–Sayre syndrome	6.7%	1
Extra-renal manifestations before transplant		
Neurological (overall)	86.7%	13
- SNHL	80.0%	12
- developmental delay	46.7%	7
- hypotonia	40.0%	6
- stroke	13.3%	2
- myopathy	13.3%	2
- others	20.0%	3
Faltering growth	60.0%	9
Endocrine	6.7%	1
Cardiovascular	33.3%	5
Others (Sideroblastic anemia)	6.7%	1
Renal manifestation		
Median age at presentation (*n* = 10)	5	(range 0.3–8)
Types of presentation		
Nephrotic syndrome	6.7%	1
Nephrotic range proteinuria	33.3%	5
Renal Fanconi	6.7%	1
CKD	20.0%	3
Kidney failure	20.0%	3
Distal RTA	26.7%	4
Others (cystic kidney disease)	6.7%	1
Histology (*n* = 4)		
FSGS	50.0%	2/4
Others	50.0%	2/4
Alport syndrome (genetically confirmed)	25.0%	1/4
TIN with significant IFTA	25.0%	1/4
Median age at kidney failure (*n* = 9)	10	(range 4–15)
Median time from renal presentation to kidney failure	6	(range 1–8)
Kidney transplantation		
Median age at transplant	10	(range 4–17)
Donor type (deceased:living) (*n* = 8)	37.5%:62.5%	(3:5)
Acute Peri-operative complication		
Mitochondrial disease–related complications (PRES)	6.7%	1
Hyperacute rejection	0%	
Outcome		
Median follow-up period	3	(range 0.5–7)
New-onset/worsened comorbidities post-transplant	26.7%	4
- Neurology	20.0%	1
- Endocrine	6.7%	3
Transplant-related complications	13.3%	2
Death censored graft survival	93.3%	14/15
Patient survival	93.3%	14/15

**Table 4:** Summary of adult patients identified from previous literature

Demographics	(*n* = 31)	
Sex	M:F	15:16
Median age at first presentation (years) (*n* = 14)	19	(range 2–36)
Median age at diagnosis of mitochondrial diseases (*n* = 20)	33	(range 25–56)
Mutations		
mtDNA mutation		
- m.3243A > G	87.1%	27
- m.3271 T > C	3.2%	1
- m.8618dup	3.2%	1
- m.13513G > A	3.2%	1
nuclear gene mutation		
- c.713A > G		
Phenotypes		
MELAS	67.7%	21
MIDD	22.6%	7
NARP Syndrome	3.2%	1
Leigh Syndrome	3.2%	1
RMND1-related disease	3.2%	1
Extra-renal manifestations before transplant		
Neurological (overall)	93.5%	29
- SNHL	71.0%	22
- cognitive impairment	16.1%	5
- stroke	16.1%	5
- myopathy	22.6%	7
- epilepsy	9.7%	3
- others	12.9%	4
Diabetes mellitus	58.0%	18
Cardiovascular	22.6%	7
Faltering growth	12.9%	4
Others	12.9%	4
Renal manifestation		
Median age (*n* = 22)	31	(range 12–44)
Types of presentation		
Nephrotic syndrome	9.7%	3
Nephrotic range proteinuria	51.6%	16
Kidney failure	32.3%	10
CKD	29.0%	9
Histology (*n* = 19)		
FSGS	78.9%	15/19
Others	21.1%	4/19
Median age at KF (*n* = 22)	36.5	(range 12–58)
Median time from renal presentation to KF	4	(range 1–22)
Kidney transplantation		
Median age at transplant	39	(range 12–59)
Donor type (deceased:living) (*n* = 26)	50.0%:50.0%	(13:13)
Acute peri-operative complication (*n* = 30)		
Mitochondrial disease–related complications	3.3%	1/30
Hyperacute rejection	0%	0/30
Outcome		
Median follow-up period	5	(range 0.5–22)
Maintenance immunosuppressant (*n* = 24)		
Double agents:triple agents	58.3%:41.7%	14:10
Prednisolone	58.3%	14/24
Anti-metabolite	50.0%	12/24
CNI	37.5%	9/24
mTOR inhibitors	25.0%	6/24
New-onset/worsened comorbidities post-transplant	58.0%	18
- Neurology	16.1%	5
- Endocrine	29.0%	9
- Cardiovascular	9.7%	3
- Others	6.5%	2
Transplant-related complications		
T-cell medicated rejection	6.5%	2
CMV viremia	3.2%	1
Death censored graft survival	96.7%	29/30
Patient survival	96.8%	30

Immunosuppressive regimens were reported in 52.2% (24/46) of patients. All were adults. Of these, 58.3% (14/24) received triple therapy including prednisolone, typically in combination with an anti-metabolite (e.g., mycophenolate mofetil), a calcineurin inhibitor (CNI) (e.g. tacrolimus or cyclosporin) and/or an mTOR inhibitor.

## Discussion

Several nuclear-encoded mutations, including defects in the CoQ biosynthesis pathway, have been reported to cause kidney failure requiring transplantation. As patients with CoQ 10 deficiency often present as isolated SRNS with a relatively favourable response to oral enzyme supplementation [12], we excluded them from our synthesis to maintain a clinically coherent cohort focused on non-remediable, multisystem primary mitochondrial diseases. Nonetheless, favourable kidney transplantation outcomes have been described in these patients [24, 43, 44]. For instance, Wang et al. and Zeng et al. reported two and three out of four patients with *COQ8B* mutations, respectively, maintaining good graft function 1–10 years post-transplant, while Park et al. described five children with *COQ6* mutations all with excellent graft function [24, 43, 44]. In contrast, the transplantation experience in other primary mitochondrial diseases, particularly those with irreversible multisystem involvement, remains limited and poorly characterised. With improved survival into adolescence and adulthood, an increasing proportion of up to 25% of patients with primary mitochondrial diseases now progress to CKD or even kidney failure [9, 19, 21]. Understanding transplant outcomes in this population is therefore crucial.

Although our primary focus was paediatric patients, we included adult cases to provide a broader clinical context. Notably, 25.8% of adults had childhood onset symptoms but were only diagnosed later, underscoring both diagnostic challenges and the lifelong continuum of mitochondrial disease manifestations. Despite genetic differences, adult data remain highly relevant for anticipating long-term outcomes and guiding transplant decisions across the lifespan.

While more than 1000 nuclear genes are implicated in mitochondrial function [45, 46], the only nuclear gene mutation identified in our review was *RMND1*. All ten patients with *RMND1* disease harboured the *c.713A* > *G* variant and presented by age 2, whereas nearly 80% of patients with point mutation *m.3243A* > *G* in *MT-TL1* presented in their twenties or thirties. This likely reflects distinct genotype–phenotype patterns among nuclear and mtDNA mutations. *RMND1* disease typically manifests in early infancy with type 4 RTA with or without hypertension mimicking pseudohypoaldosteronism and progresses rapidly to kidney failure during childhood [39, 41, 47]. By contrast, in other nuclear gene–associated primary mitochondrial diseases (e.g. complex I deficiencies, infantile encephalomyopathies and cardiomyopathies), children often present with severe, early-onset multisystem dysfunction predominantly affecting neurological, cardiac and hepatic systems. These more severe extra-renal manifestations and high disease mortality likely precluded most affected children from kidney transplantation [2, 3, 48, 49]. Patients with m.3243A > G in *MT-TL1* more often present in late adolescence or early adulthood with heavy proteinuria and progress more gradually until advanced CKD is reached [50, 51]. These differences reflect both intrinsic disease course and referral thresholds for transplantation.

More than 80% of patients with renal involvement also had neurological features, highlighting the systemic nature of primary mitochondrial diseases. Faltering growth, present in 60% of the cohort, is another important common feature. At initial nephrology referral, an overall of 26.1% (*n* = 12/46) of patients had already progressed to kidney failure, while 26.1% (*n* = 12/46) were in different stages of CKD. Among those not yet in kidney failure, the median time from first renal presentation to progression was just 6 years. Bakis et al. reported 35% of their patients with mitochondrial disease as suffering from stage 2 or higher CKD [52]. These data emphasise the need for early renal surveillance in patients with primary mitochondrial diseases.

In our review, death-censored graft survival and patient survival rates were above 90% across patient groups with different genetic variants and age groups. By comparison, recent registries report 5-year paediatric graft and patient survival of 85–95% and 96–97%, respectively [53–55], and adult rates of 80–90% and 90–95% [46, 55]. While numbers are small and subject to selection bias, our findings suggest that outcomes in primary mitochondrial diseases are broadly comparable to those with more common causes of kidney failure undergoing transplantation.

Several factors may account for these encouraging results. First, early and accurate molecular diagnosis now shortens the diagnostic odyssey, supports anticipatory management and informs transplant timing. Genetic confirmation also prompts targeted screening for subclinical extra-renal involvement, such as dilated cardiomyopathy in *RMND1* mutations, which could otherwise preclude transplantation [21, 41]. It further clarifies inheritance patterns. Mitochondrial disease is not invariably maternally inherited. Autosomal recessive and dominant forms also exist, especially with nuclear encoded gene mutations which are more prevalent in the paediatric population [56, 57]. This distinction is crucial for family counselling and donor selection. In our review, three children and an adult with autosomal recessive primary mitochondrial diseases received living related grafts from carrier parents and maintained excellent graft function.

Secondly, growing awareness of mitochondrial pathophysiology has refined multidisciplinary peri-operative care involving metabolic physicians, dieticians, specialised nurses, anaesthetists and nephrologists. The metabolic team outlines a detailed pre-, peri- and post-operative monitoring plan for glucose, lactate and acid–base balance and provides expert input on medications which are best avoided due to mitochondrial toxicity. A proposed approach for perioperative management of kidney transplant recipients with metabolic conditions has been recently published [58]. Anaesthetic agents with recognised mitochondrial risk, such as prolonged propofol infusions and succinylcholine, are routinely avoided [59]. Intra-operative monitoring has also become more targeted, with close surveillance of metabolic parameters. In selected cases, such as patients with MELAS, intravenous arginine is also administered peri-operatively to reduce the risk of stroke-like episodes [60].

Thirdly, improvements in immunosuppressive regimens have likely contributed to better transplant outcomes in mitochondrial disease. The widespread adoption of mycophenolate combined with tacrolimus has been associated with lower rejection rates and improved graft survival in kidney transplantation overall [61–63]. Importantly, despite the theoretical risks of tacrolimus in inducing mitochondrial dysfunction through mechanisms like disrupting calcium signalling, adult data in this review suggest this combination is generally well tolerated without significant metabolic decompensation [64–66]. mTOR inhibitors such as everolimus have been proposed as potentially metabolically safer alternatives [30, 67]. However, current evidence remains anecdotal and insufficient to support routine substitution over established protocols such as CNIs, including tacrolimus and, in some cases, cyclosporin A.

Beyond graft survival, broader indicators of transplant success must be considered. Overall, post-transplant progression of underlying disease was observed in 56.5% of patients (26/46) mainly in terms of neurological deterioration or worsened glycaemic control. These findings highlight the need for long-term, multisystem monitoring and integrated post-transplant care. However, functional outcomes and quality of life are rarely reported. Incorporating standardised pre- and post-transplant assessments would better guide rehabilitation and support shared decision-making regarding long-term care.

This study has several strengths. To our knowledge, it is the most comprehensive synthesis to date examining kidney transplantation in primary mitochondrial diseases. We included both paediatric and adult cases to provide a broad, age-spanning clinical perspective, and systematically analysed outcomes across different genotypes. Only patients with genetically confirmed diagnoses and available post-transplant data were included, strengthening the diagnostic accuracy and outcome reliability of the review. By synthesising existing evidence, we were able to highlight key trends across age groups and genetic backgrounds.

However, several limitations must be acknowledged. As mentioned above, this review is based predominantly on case reports and small case series, which are inherently subject to small sample size, selection bias and reporting bias. Publication bias is also possible, as favourable outcomes are more often reported. Considerable heterogeneity exists in the depth of clinical detail, follow-up duration and outcome measures across studies, limiting the comparability of cases. While we excluded reports lacking genetic confirmation or post-transplant follow-up, the retrospective nature of most studies and the absence of standardised functional or quality-of-life assessments remain notable limitations.

In summary, with careful patient selection, multidisciplinary management and individualised transplant assessments, kidney transplantation represents a viable treatment option for mitochondrial disease-associated kidney failure with good reported patient and graft outcomes. Patients with mitochondrial disease suffering from kidney failure should be considered and assessed for kidney transplantation. Future research integrating systematic quality-of-life assessments is essential to better characterise long-term outcomes and address post-transplant disease progression.

## Supplementary Information

Below is the link to the electronic supplementary material.

ESM1 Graphical abstract (PPTX 136 KB)

ESM2 (DOCX 30.2 KB)

ESM3 (XLSX 13.7 KB)

ESM4 (XLSX 14.3 KB)

## Data Availability

All data generated or analysed during this study are included in this published article and its supplementary information files.
