# Supplementary material for: Kidney transplantation in mitochondrial diseases: a systematic review
Source: Pediatr Nephrol. 2025 Nov 27;41(8):2443–55. doi: 10.1007/s00467-025-07034-3 (PMC13337731; doi:10.1007/s00467-025-07034-3)
Supplement: Supplementary file 1 — Graphical abstract (PPTX 136 KB) [file 467_2025_7034_MOESM1_ESM.pptx]

## Slide 1
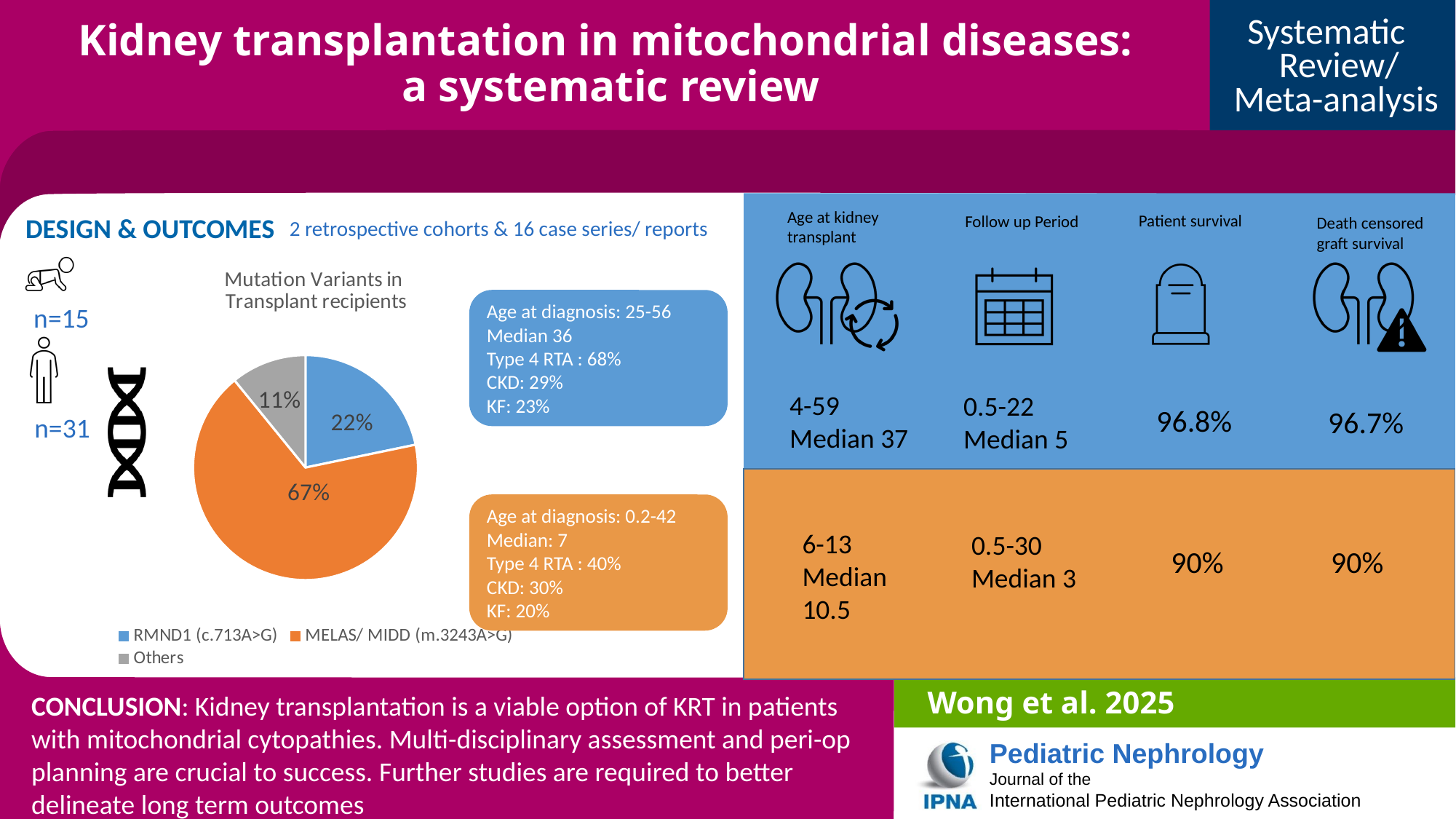

Kidney transplantation in mitochondrial diseases:
a systematic review
2 retrospective cohorts & 16 case series/ reports
Age at kidney transplant
Patient survival
Follow up Period
DESIGN & OUTCOMES
Death censored graft survival
n=15
n=31
### Chart: Mutation Variants in
Transplant recipients
| Category | |
|---|---|
| RMND1 (c.713A>G) | 0.21739130434782608 |
| MELAS/ MIDD (m.3243A>G) | 0.6739130434782609 |
| Others | 0.10869565217391304 |
Age at diagnosis: 25-56
Median 36
Type 4 RTA : 68%
CKD: 29%
KF: 23%
4-59
Median 37
0.5-22
Median 5
96.8%
96.7%
Age at diagnosis: 0.2-42
Median: 7
Type 4 RTA : 40%
CKD: 30%
KF: 20%
6-13
Median 10.5
0.5-30
Median 3
90%
90%
Wong et al. 2025
CONCLUSION: Kidney transplantation is a viable option of KRT in patients with mitochondrial cytopathies. Multi-disciplinary assessment and peri-op planning are crucial to success. Further studies are required to better delineate long term outcomes
