# Supplementary material for: Kidney transplantation in mitochondrial diseases: a systematic review
Source: Pediatr Nephrol. 2025 Nov 27;41(8):2443–55. doi: 10.1007/s00467-025-07034-3 (PMC13337731; doi:10.1007/s00467-025-07034-3)
Supplement: Supplementary file 2 — (DOCX 30.2 KB) [file 467_2025_7034_MOESM2_ESM.docx]

**Supplementary Figure 1** Search strategy for publication identification

1. Mitochondrial disorder*
2. Mitochondrial disease*
3. Mitochondrial cytopath*
4. “Mitochondriopath*”
5. MELAS
6. MIDD
7. RMND1
8. Leigh
9. 1 OR 2 OR 3 OR 4 OR 5 OR 6 OR 7 OR 8
10. kidney OR Renal
11. Transplants [Mesh]
12. Transplantation [Mesh]
13. 11 OR 12
14. Outcome*
15. 9 AND 10 AND 13 AND 14

Supplemental Table 1. Excluded studies

| Studies | Reason for exclusion |
| --- | --- |
| Montenegro et al. [1]  Anderson et al. [2] | Multi-organ transplant was involved |
| Kim et al. [3]  Lim et al. [4] | Post-transplant follow-up data were not available |

Reference

1. Arriola-Montenegro J, Mutschler M, Cogswell R, Alexy T, John R, Voeller R, Humphreville V, Aggarwal A, Maharaj V (2024) Successful Simultaneous Heart-Kidney Transplant in a Patient With *MT-TL1* MELAS Cardiomyopathy. JACC: Case Reports 29:102523. https://doi.org/10.1016/j.jaccas.2024.102523

2. Anderson E, Setty S, Dahmen M, Townsley MM, Augoustides JG, Fernando RJ (2025) Cardiac and Renal Transplantation in Mitochondrial Encephalopathy, Lactic Acidosis, and Stroke-like Symptoms: Anesthetic Challenges and Considerations. Journal of Cardiothoracic and Vascular Anesthesia 39:301–308. https://doi.org/10.1053/j.jvca.2024.09.138

3. Kim JH, Kim D, Hwang S, Kim G-H, Lee BH, Yoo H-W, Choi J-H (2025) Endocrine manifestations and long-term outcomes of patients with mitochondrial diseases. Orphanet J Rare Dis 20. https://doi.org/10.1186/s13023-025-03773-6.

4. Lim K, Steele D, Fenves A, Thadhani R, Heher E, Karaa A (2017) Focal segmental glomerulosclerosis associated with mitochondrial disease. CNCS 5:20–25. https://doi.org/10.5414/CNCS109083

Supplemental Table 2A. Quality Assessment for Included Case Reports/ Series

| Studies | Domains | | | | | | | Overall Quality |
| --- | --- | --- | --- | --- | --- | --- | --- | --- |
|  | Comprehensive  Selection | Well defined Exposure | | Well defined Outcome | Alternative Causes has been Ruled Out | Adequate  Follow-up | Detailed Reporting |  |
| Ravn et al.[20] | Unclear | Yes | Yes | | Yes | Unclear | Yes | Fair |
| Roper et al.[22] | Unclear | Yes | Yes | | Yes | Yes | Yes | Fair |
| Hameed et al.[27] | Yes | Yes | Yes | | Yes | Yes | Yes | Good |
| Lederer et al.[28] | Yes | Yes | Yes | | Yes | Yes | Yes | Good |
| Johnson et al.[29] | Yes | Yes | Yes | | Yes | Yes | Yes | Good |
| Laat et al.[30] | Yes | Yes | Yes | | Yes | Yes | Yes | Good |
| Szczepanik et al.[31] | Yes | Yes | Yes | | Yes | No | Yes | Fair |
| Ducharlet et al.[32] | Unclear | Yes | Yes | | Yes | Yes | Yes | Fair |
| Sousa et al.[33] | Yes | Yes | Yes | | Yes | Yes | Yes | Good |
| Guéry et al.[34] | Yes | Yes | Yes | | No | No | Unclear | Poor |
| Nishida et al.[35] | Yes | Yes | Yes | | Yes | Yes | Yes | Good |
| Broenen et al.[37] | Yes | Yes | Yes | | No | Yes | Yes | Fair |
| Shayota et al.[38] | Yes | Yes | Yes | | No | Unclear | Yes | Fair |
| Kömhoff et al.[39] | Yes | Yes | Yes | | Yes | Yes | Unclear | Fair |
| Seidowsky et al.[41] | Unclear | Yes | Yes | | No | Yes | Unclear | Poor |
| Stein et al.[42] | Yes | Yes | Yes | | No | Yes | Yes | Fair |

Supplemental Table 2B. BMJ AXIS Tool Assessment for Retrospective Cohort

|  | Ng et al. [40] | Parikh et al.[36] |
| --- | --- | --- |
| Introduction |  |  |
| Were the aims/objectives of the study clear? | Yes | Yes |
| Method |  |  |
| Was the study design appropriate for the stated aim(s)? | Yes | Yes |
| Was the sample size justified? | Not applicable | Not applicable |
| Was the target/reference population clearly defined? (Is it clear who the research was about?) | Yes | Yes |
| Was the sample frame taken from an appropriate population base so that it closely represented the target/reference population under investigation? | Yes | Yes |
| Was the selection process likely to select subjects/participants that were representative of the target/reference population under investigation? | Yes | Yes |
| Were measures undertaken to address and categorise non-responders? | Not applicable | Not applicable |
| Were the risk factor and outcome variables measured appropriate to the aims of the study? | Yes | Yes |
| Were the risk factor and outcome variables measured correctly using instruments/measurements that had been trialled, piloted or published previously? | Yes | Yes |
| Is it clear what was used to determined statistical significance and/or precision estimates? (eg, p values, CIs) | Not applicable |  |
| Were the methods (including statistical methods) sufficiently described to enable them to be repeated? | Yes | Yes |
| Results |  |  |
| Were the basic data adequately described? | Yes | Yes |
| Does the response rate raise concerns about non-response bias? | Not applicable | Not applicable |
| If appropriate, was information about non-responders described? | Not applicable | Not applicable |
| Were the results internally consistent? | Yes | Yes |
| Were the results for the analyses described in the methods, presented? | Yes | Yes |
| Discussion |  |  |
| Were the authors’ discussions and conclusions justified by the results? | Yes | Yes |
| Were the limitations of the study discussed? | Yes | Yes |
| Other |  |  |
| Were there any funding sources or conflicts of interest that may affect the authors’ interpretation of the results? | Yes | Yes |
| Was ethical approval or consent of participants attained? | Yes | Yes |
